# BCL-X_L_ inhibitors enhance the apoptotic efficacy of BRAF inhibitors in *BRAF*^*V600E*^ colorectal cancer

**DOI:** 10.1038/s41419-024-06478-z

**Published:** 2024-03-01

**Authors:** Laura J. Jenkins, Ian Y. Luk, Fiona Chionh, Tao Tan, Kristen Needham, Jamieson Ayton, Camilla M. Reehorst, Natalia Vukelic, Oliver M. Sieber, Dmitri Mouradov, Peter Gibbs, David S. Williams, Niall C. Tebbutt, Jayesh Desai, Frédéric Hollande, Amardeep S. Dhillon, Erinna F. Lee, Delphine Merino, W. Douglas Fairlie, John M. Mariadason

**Affiliations:** 1 Olivia Newton-John Cancer Research Institute, Melbourne, VIC Australia; 2 School of Cancer Medicine, La Trobe University, Melbourne, VIC Australia; 3 Personalised Oncology Division, The Walter and Eliza Hall Institute of Medical Research, Melbourne, VIC Australia; 4 Department of Medical Biology, The University of Melbourne, Melbourne, VIC Australia; 5 Department of Surgery, The University of Melbourne, Melbourne, VIC Australia; 6 Department of Biochemistry and Molecular Biology, Monash University, Melbourne, VIC Australia; 7 Department of Pathology, Austin Health, Melbourne, VIC Australia; 8 Department of Medical Oncology, Austin Health, Melbourne, Australia; 9 Peter MacCallum Cancer Centre, Melbourne, VIC Australia; 10 Department of Clinical Pathology, The University of Melbourne, Melbourne, VIC Australia; 11 University of Melbourne Centre for Cancer Research, Victorian Comprehensive Cancer Centre, Melbourne, VIC Australia; 12 The institute for Mental and Physical Health and Clinical Translation, School of Medicine, Deakin University, Geelong, Australia; 13 Department of Biochemistry and Chemistry, School of Agriculture, Biomedicine and Environment, La Trobe Institute for Molecular Science, La Trobe University, Melbourne, VIC Australia; 14 Immunology Division, The Walter and Eliza Hall Institute of Medical Research, Melbourne, VIC Australia

**Keywords:** Colon cancer, Targeted therapies

## Abstract

Metastatic *BRAF*^*V600E*^ colorectal cancer (CRC) carries an extremely poor prognosis and is in urgent need of effective new treatments. While the BRAF^V600E^ inhibitor encorafenib in combination with the EGFR inhibitor cetuximab (Enc+Cet) was recently approved for this indication, overall survival is only increased by 3.6 months and objective responses are observed in only 20% of patients. We have found that a limitation of Enc+Cet treatment is the failure to efficiently induce apoptosis in *BRAF*^*V600E*^ CRCs, despite inducing expression of the pro-apoptotic protein BIM and repressing expression of the pro-survival protein MCL-1. Here, we show that *BRAF*^*V600E*^ CRCs express high basal levels of the pro-survival proteins MCL-1 and BCL-X_L_, and that combining encorafenib with a BCL-X_L_ inhibitor significantly enhances apoptosis in *BRAF*^*V600E*^ CRC cell lines. This effect was partially dependent on the induction of BIM, as BIM deletion markedly attenuated BRAF plus BCL-X_L_ inhibitor-induced apoptosis. As thrombocytopenia is an established on-target toxicity of BCL-X_L_ inhibition, we also examined the effect of combining encorafenib with the BCL-X_L_ -targeting PROTAC DT2216, and the novel BCL-2/BCL-X_L_ inhibitor dendrimer conjugate AZD0466. Combining encorafenib with DT2216 significantly increased apoptosis induction in vitro, while combining encorafenib with AZD0466 was well tolerated in mice and further reduced growth of *BRAF*^*V600E*^ CRC xenografts compared to either agent alone. Collectively, these findings demonstrate that combined BRAF and BCL-X_L_ inhibition significantly enhances apoptosis in pre-clinical models of *BRAF*^*V600E*^ CRC and is a combination regimen worthy of clinical investigation to improve outcomes for these patients.

## Introduction

Mutations in the *BRAF* oncogene (predominantly *BRAF*^*V600E*^) occur in approximately 10% of metastatic CRCs and drive tumorigenesis by constitutive activation of the MAPK/ERK signalling pathway [1–4]. In metastatic CRC, *BRAF*^*V600E*^ mutations are associated with a particularly poor prognosis [5–7].

Despite inducing objective responses in ~50% of melanoma patients [8], monotherapy using BRAF inhibitors such as vemurafenib, dabrafenib and encorafenib only induce objective responses in ~5% of *BRAF*^*V600E*^ metastatic CRCs [9]. This was attributed to relief of a negative feedback loop between ERK and the EGFR, leading to re-activation of MAPK/ERK signalling [10, 11]. Based on this observation, combination treatment with encorafenib (Enc) and the EGFR inhibitor cetuximab (Cet) were tested and found to significantly improve overall survival in *BRAF*^*V600E*^ metastatic CRC patients [12, 13], and is the current standard of care for these patients. While the Enc+Cet regimen represents a major advance in the treatment of *BRAF*^*V600E*^ CRC, the improvement in overall survival (OS) is <4 months and objective response rates remain below 20% [13], warranting the need for further refinement of this treatment.

One limitation of BRAF and MAPK/ERK pathway inhibitors in general is their failure to induce substantial levels of apoptosis in CRC and other tumour cell types [11, 14]. Notably, this is despite these agents inducing the expression of the pro-apoptotic proteins BIM [15–17], BMF [17] and PUMA [15, 17], and repressing expression of the pro-survival protein MCL-1 [16, 18].

Notably, CRC cells have been reported to express high basal levels of pro-survival proteins, particularly BCL-X_L_ [19]. Herein, we considered the possibility that the high basal level of expression of pro-survival proteins may establish a high apoptotic threshold in CRC cells that cannot be overcome by BRAF inhibitors alone. To overcome this, we investigated the combinatorial use of BRAF inhibitors with BH3 mimetics, a class of anti-cancer compounds that mimic the function of BH3-only proteins to block the activity of pro-survival proteins [20]. We found that both MCL-1 and BCL-X_L_ are highly expressed in *BRAF*^*V600E*^ CRC cell lines and that combining BRAF inhibitors with BCL-X_L_ inhibitors significantly enhances apoptosis in these cell lines. We also demonstrate that this effect is at least partially dependent on the induction of BIM. Importantly, we further confirm this finding using two emerging strategies of BCL-X_L_ inhibition, first using the BCL-X_L_ targeting PROTAC DT2216 and second using the novel BCL-2/BCL-X_L_ inhibitor dendrimer conjugate AZD0466 [21]. Our pre-clinical findings demonstrate that the addition of BCL-X_L_ inhibitors to BRAF inhibitor treatment is worthy of clinical investigation as a means of improving outcomes for *BRAF*^*V600E*^ CRC patients.

## Methods

### Cell culture

The *BRAF*^*V600E*^ colorectal cancer cell lines were sourced as follows; COLO 201, COLO 205, HT29, SW1417 and RKO (American Type Culture Collection, Manassas, VA, USA), LIM2551 and LIM2405 cells (Ludwig Institute for Cancer Research), CO115 [22], VACO432 [23], LS411 [24] and VACO5 [25]. All cell lines were maintained in Dulbecco’s Minimal Essential Media/F12 (DMEM/F12, Thermo Fisher Scientific, Waltham, MA, USA) supplemented with 5% FBS (v/v)(Moregate, Queensland, Australia) at 37 °C with 5% CO_2_. Cell line authentication was performed by short tandem repeat (STR) profiling using the GenePrint 10 system (Promega, Madison, WI, USA), and all lines were found to be exact matches to published profiles. Mycoplasma testing was performed every 3-6 months as part of routine monitoring in our laboratory.

### Chemicals

Encorafenib, vemurafenib, A-1331852, S63845, ABT-199 (all from Assay Matrix, Melbourne, Australia), DT2216 (Axon Medchem, Netherlands) and AZD4320 (AstraZeneca, Cambridge, UK) were dissolved in dimethyl sulfoxide (DMSO, Sigma-Aldrich, St. Louis, MO, USA). Cetuximab (Erbitux, Lilly, Indianapolis, IN, USA) was obtained from the Austin Health Pharmacy. AZD0466 was obtained from AstraZeneca.

### Western blot

Whole cell lysates were prepared using NP-40 lysis buffer (50 mM Tris-HCL pH 7.5, 150 mM NaCl, 1% NP-40 (v/v), 1 mM EDTA pH 8) supplemented with cOmplete protease inhibitor and PhosSTOP phosphatase inhibitor cocktails (Roche, Basel, Switzerland). A total of 30 μg of protein per sample was resolved on NuPAGE 4-12% Bis-Tris pre-cast polyacrylamide gels (Novex, Thermo Fisher Scientific), transferred onto iBLOT2 Polyvinylidene Difluoride (PVDF) membranes (Invitrogen, Thermo Fisher Scientific) and blocked using Odyssey blocking buffer (LiCor, Lincoln, NE, USA). Primary antibodies used in western blot analysis were BIM_S/L/EL_ (ALX-804-527, Enzo Life Sciences, New York, NY, USA), MCL-1 (5453S, Cell Signalling Technologies, Danvers, MA, USA), BCL-X_L_ (2764, Cell Signalling Technologies), Cleaved Caspase 3 (9661, Cell Signalling Technologies), and β-tubulin (ab6046, Abcam, Cambridge, UK). Secondary antibodies used were IRDye®680RD Goat anti-rat IgG (H + L)(926-68076, LiCor) and IRDye®800CW Goat anti-rabbit IgG (H + L)(926-32211, LiCor). Full western blot images can be found in the Supplementary File.

### Flow cytometry

Cells were seeded in 24 well plates and treated with drugs for 24 or 72 h. Both adherent and non-adherent cells were collected at completion of drug treatment and incubated overnight at 4 °C in 50 μg/mL propidium iodide diluted in sodium citrate buffer (0.1% sodium citrate (w/v) and 0.1% Triton X-100 (v/v)) (all from Sigma-Aldrich). Samples were then analysed on a BD FACSymphony A3 flow cytometer (BD Biosciences, Franklin Lakes, NJ, USA), by analysis of 10,000 events. Apoptotic cells were defined as having a sub-diploid DNA content and quantified using FlowJo V8.0 (FlowJo LLC, Ashland, OR, USA). Cell cycle analysis was performed using ModFit LT^TM^ version 2.0 (Verity Software House, Topsham, ME, USA).

### Clonogenic survival assays

Cells were seeded at 500 cells per well in 12-well plates and treated the following day with encorafenib (1 nM or 100 nM) alone and in combination with either A-1331852 (10 nM), S63845 (1 μM) or ABT-199 (1 μM). Colonies were then allowed to form over 10 days, at this point cells were fixed with 10% formalin for 5 min and stained with 0.1% crystal violet solution (Sigma-Aldrich) for 15 min at room temperature, washed with PBS and allowed to air dry. Analysis of clonogenic survival was performed using ImageJ (Java).

### Generation of BIM knockout COLO 201 and LIM2551 cells

The COLO 201 and LIM2551 BIM knockout cell line was generated using the previously described doxycycline-inducible CRISPR-Cas9 lentiviral system [26]. Briefly, cells were stably transduced with lentivirus expressing Cas9-mCherry and FACS sorted for mCherry positivity. Cells were subsequently transduced with lentivirus expressing GFP and the doxycycline-inducible sgRNA sequence targeting exon 3 of the human *BIM* gene (5′-GCCCAAGAGTTGCGGCGTAT-3′). Cells were double sorted for mCherry and GFP and subsequently maintained in vehicle or doxycycline (1 μg/ml).

### Xenograft study

Six-week-old female NOD Scid gamma (NSG) mice were obtained from a colony maintained at the Austin Health BioResource Facility (Melbourne, Australia) and housed in specific pathogen free (SPF) microisolators. Mice were subcutaneously injected with 2 × 10^6^ COLO 201 cells into the left and right flanks in a 1:1 mixture of matrigel matrix (75 μL)(Corning): DMEM/F12 (75 μL). Once tumours became palpable (~100 mm^3^), mice were randomised to receive either vehicle control (*n* = 8 mice, 16 tumours), encorafenib (20 mg/kg, bi-daily, oral gavage) (*n* = 8 mice, 16 tumours), AZD0466 (103 mg/kg, once weekly, tail vein injection) (*n* = 8 mice, 16 tumours) or encorafenib plus AZD0466 (*n* = 8 mice, 14 tumours) for a total of 3 weeks. Tumours from one mouse were excluded from the combination arm due to an adverse event unrelated to treatment. Allocation of mice into groups was not blinded by the investigator. Sample sizes of the cohorts were based on previous experience, however no formal power analyses were performed prior to experimentation. Calliper measurements blinded to treatment group were performed a minimum of three times per week to measure tumour size. Investigators were not blinded at endpoint for tumour collection. Encorafenib was prepared in 50% PhosalPG (v/v), 27.5% PEG400 (v/v), 10% Ethanol (v/v) and 2.5% DMSO (v/v). AZD0466 was prepared in citrate/phosphate buffer. The study was approved by the Austin Health Animal Ethics Committee (A2018_05584).

### Microarray analysis

Gene expression profiling was performed on 11 *BRAF*-mutant colorectal cancer cell lines treated with vemurafenib (5 μM) or DMSO control for 6 h, using the Affymetrix HG-U-133-Plus-2-Human Genome Array platform at the Peter MacCallum Cancer Centre genomics core facility (Melbourne, Australia). Affymetrix microarray. CEL files were imported into the Partek Genomics Suite version 6.6 (Partek, Chesterfield, MO, USA) and normalised using the Robust Multiarray Averaging (RMA) method. Normalised data was analysed using the Limma package. Affymetrix probes were determined to be differentially expressed between DMSO control and vemurafenib treatments if the adjusted *p-*value was <0.05.

### Immunohistochemistry

Formalin-fixed paraffin-embedded sections (4 μm) were de-paraffinized and rehydrated through serial washes in xylene and ethanol. Sections were rinsed in H_2_O and quenched in 3% H_2_O_2_ (Chemsupply) for 10 min. Antigen retrieval was performed by heating in Citrate buffer (pH 6.0) for 2 min at 1.0 power and 8 min at 0.2 power in the microwave. Slides were probed and incubated with anti-BIM (1:100, Cell Signalling Technology, 2933S) primary antibody at 4 °C overnight. Slides were then washed and incubated with Labeled polymer HRP-anti Rabbit (K4003, Dako) for 1-h at room temperature. Slides were washed and chromagen was developed using the DAB (3, 3-diaminobenzide) reagent (Dako). Sections were counter-stained using pre-filtered Mayer’s hematoxylin (Amber Scientific, Australia) then dehydrated through serial ethanol and xylene washes prior to mounting using DPX mounting solution (Sigma-Aldrich). BIM expression was analysed using HALO software (Indica Labs, NM, USA).

### Statistics

Statistical analyses were performed using GraphPad Prism v8.0 software (GraphPad Software, La Jolla, CA, USA). Groups were compared using either Student’s *t* test with Welch’s correction or One-way ANOVA with Tukey’s multiple comparison, unless stated otherwise. In all cases: ns (not significant), *(*p* ≤ 0.05), **(*p* ≤ 0.01), ***(*p* ≤ 0.001) and ****(*p* ≤ 0.0001).

## Results

### BRAF inhibitors inhibit proliferation but have minimal effects on apoptosis in *BRAF*^*V600E*^ CRC cell lines

**Fig. 1:**
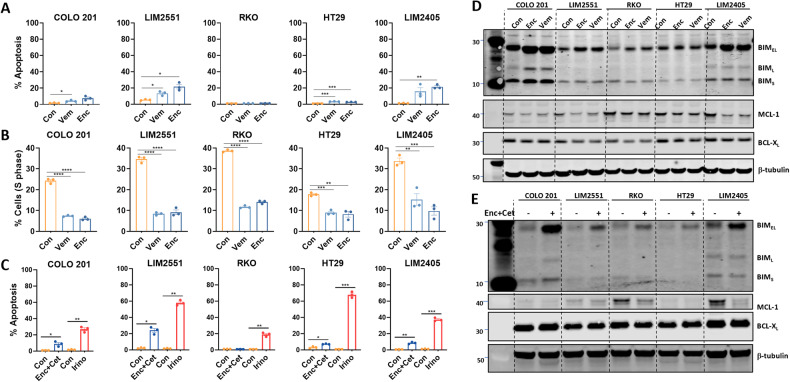
BRAF inhibitors induce predominantly cytostatic effects in *BRAF*^*V600E*^ CRC cell lines. **A, B** *BRAF*^V600E^ CRC cell lines were treated with vemurafenib (Vem, 5 μM) or encorafenib (Enc, 100 nM) for 72 h and stained with propidium iodide (PI) and analysed by FACS to determine (**A**) apoptosis induction (percentage of cells with sub-diploid DNA content), and (**B**) percentage of cells in S-phase. Values shown are mean ± SEM from a representative experiment performed in technical triplicate. Similar results were obtained in two additional independent experiments. Groups were compared using an unpaired Student’s t-test using Welch’s correction; *(*p* ≤ 0.05), **(*p* ≤ 0.01) and ***(*p* ≤ 0.001). **C** *BRAF*^*V600E*^ CRC cell lines were treated with encorafenib (100 nM) plus cetuximab (10 μg/mL) (Enc+Cet) or irinotecan (10 μM)(Irino) for 72 h and apoptosis levels determined by PI staining as above. Values shown are mean ± SEM from a representative experiment performed in technical triplicate. Similar results were obtained in 2 additional independent experiments. Differences were compared using one-way ANOVA with Tukey’s multiple comparison testing; ns (not significant), *(*p* ≤ 0.05), **(*p* ≤ 0.01) and ****(*p* ≤ 0.0001). **D**, **E** Western blot analysis for BIM_S/L/EL_, MCL-1 and BCL-X_L_ in *BRAF*^*V600E*^ CRC cell lines treated with (**D**) encorafenib (100 nM) or vemurafenib (5 μM) or (**E**) encorafenib (100 nM) plus cetuximab (10 μg/mL) for 6 h. β-tubulin was used as a loading control.

BRAF inhibitor monotherapy induces objective responses in only ~5% of metastatic *BRAF*^*V600E*^ CRC patients [9]. While combination BRAF plus EGFR inhibitor treatment improves this to ~20%, overall survival is increased by only 4 months. One limitation of MAPK pathway inhibitors in general is that they predominantly induce cytostatic effects, and often fail to induce extensive levels of apoptosis [11, 14]. To investigate if similar effects occur upon BRAF inhibitor treatment of *BRAF*^*V600E*^ CRC cells, we treated five *BRAF*^*V600E*^ CRC cell lines with the BRAF inhibitors vemurafenib or encorafenib. Both inhibitors induced minimal to modest (<25%) apoptosis across all cell lines (Fig. 1A). Comparatively, both agents inhibited cell cycle progression in all five *BRAF*^*V600E*^ CRC lines tested, illustrated by the significant reduction in the percentage of cells in S-phase and increase in the percentage of cells in the G0-G1 phase (Fig. 1B, Supplementary Fig. 1).

To determine if the limited effect of BRAF inhibitors on apoptosis extended to the clinically relevant encorafenib+cetuximab (Enc+Cet) combination, we treated the five *BRAF*^*V600E*^ CRC cell lines with Enc+Cet. As observed for single agent BRAF inhibitor treatment, the combination regimen also induced minimal to modest (<25%) apoptosis across all cell lines (Fig. 1C), contrasting with the effect of the chemotherapeutic agent, irinotecan, which induced >50% apoptosis in two of five lines and >25% apoptosis in the remaining cell lines (Fig. 1C).

### BRAF inhibitors prime *BRAF*^*V600E*^ CRC cells for apoptosis

Inhibition of MAPK/ERK signalling has been shown to alter the apoptotic rheostat in tumour cells by inducing expression of several pro-apoptotic proteins and suppressing expression of several pro-survival proteins of the intrinsic apoptotic pathway [15, 27]. To determine if BRAF inhibitors induce similar effects in *BRAF*^*V600E*^ CRC cells, we profiled gene expression changes in 12 *BRAF*^*V600E*^ CRC cell lines treated with vemurafenib. As expected, vemurafenib treatment significantly reduced expression of the MAPK target genes *FOSL1*, *FOS*, *SPRY2* and *DUSP4* (Supplementary Fig. 2). Vemurafenib also significantly induced expression of the pro-apoptotic gene *BCL2L11* (BIM) and repressed expression of the pro-survival gene *MCL1*, as well as a more modest suppression of *BCL2L1* (BCL-X_L_) (Supplementary Fig. 2). Expression of the pro-apoptotic gene *BID* and the apoptosis effector genes *BAK1* and *BOK* were also downregulated by vemurafenib treatment, although the magnitude of repression was relatively modest. The induction of BIM and repression of MCL-1 were further confirmed at the protein level in the majority of the *BRAF*^*V600E*^ CRC cell lines tested (Fig. 1D, Supplementary Fig. 2). The induction of BIM and suppression of MCL-1 protein was also observed following treatment with Enc+Cet (Fig. 1E). Collectively, these findings suggest that despite their relatively modest effects on apoptosis, BRAF inhibitors may prime *BRAF*^*V600E*^ CRC cell lines to undergo apoptosis by altering the apoptotic rheostat.

### *BRAF*^*V600E*^ CRCs express high levels of MCL-1 and BCL-X_L_

**Fig. 2:**
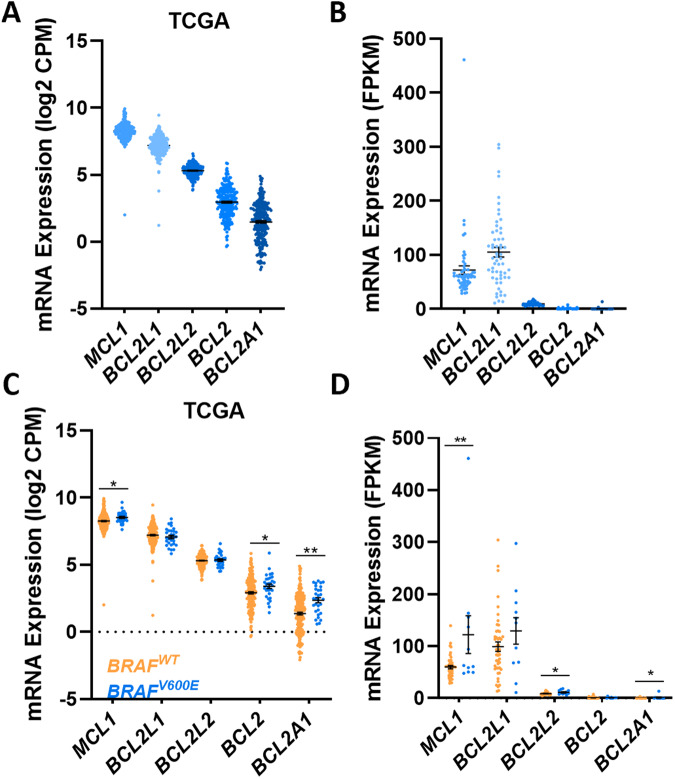
CRCs express high basal expression of BCL-X_L_ and MCL-1. **A** Basal mRNA expression levels of pro-survival genes in human CRCs. Data obtained from the COAD cohort profiled by the cancer genome atlas (TCGA)(*n* = 266). **B** Basal mRNA expression of pro-survival genes in CRC cell lines. mRNA expression was extracted from *n* = 58 CRC cell lines profiled by RNA-seq analysis as previously reported [29]. **C** Relative mRNA expression of pro-survival genes in primary *BRAF*^*WT*^ versus *BRAF*^*V600E*^ CRCs. Data obtained from the TCGA COAD cohort (WT, *n* = 236, *BRAF*-mutant, *n* = 30). **D** Relative mRNA expression of pro-survival genes in *BRAF*^*WT*^ versus *BRAF*^*V600E*^ CRC cell lines. mRNA expression was extracted from *n* = 47 *BRAF*^*WT*^ and *n* = 11 *BRAF*^*V600E*^ CRC cell lines profiled by RNA-seq analysis by [29].

As apoptosis is regulated by the balance of expression between pro-apoptotic and pro-survival proteins, we postulated that the magnitude of BIM induction and MCL-1 repression induced by BRAF inhibition may be insufficient to trigger apoptosis (BAX/BAK activation), if CRC cells express high levels of one or more pro-survival proteins. To investigate this, we interrogated the TCGA COAD RNA-seq dataset [28], as well as RNA-seq data from a large panel of CRC cell lines [29] to examine the basal expression levels of the major pro-survival proteins in CRCs. These analyses revealed higher expression of *MCL1* and *BCL2L1* compared to *BCL2*, *BCL2L2* and *BCL2A1* in both primary CRCs (Fig. 2A) and CRC cell lines (Fig. 2B). Analysis of the *BRAF*^*V600E*^ subset of primary CRCs and CRC cell lines within these datasets also revealed the same overall trend of high basal mRNA expression of *MCL1* and *BCL2L1* compared to other pro-survival proteins (Fig. 2C, D, blue). Furthermore, comparison of *BRAF*^*V600E*^ versus *BRAF*^*WT*^ CRCs in these datasets revealed that *MCL1* mRNA expression was also significantly higher in *BRAF*^*V600E*^ tumours (Figs. 2C and 2D, blue**)**. This finding suggests that MCL-1 is further upregulated by mutant BRAF signalling, which is also consistent with the repression of MCL-1 following BRAF inhibitor treatment (Fig. 1D, S2). *BCL2* and *BCL2A1* levels were also higher in *BRAF*^*V600E*^ compared to *BRAF*^WT^ tumours (Fig. 2C, D, blue), although the overall level of expression was considerably lower than that of *MCL1* and *BCL2L1*. Collectively these findings reveal that BCL-X_L_ and MCL-1 are the most highly expressed pro-survival factors expressed in *BRAF*-mutant CRCs, and that MCL-1 is further upregulated in *BRAF*^*V600E*^ tumours.

### Combining BRAF inhibitors with a BCL-X_L_ inhibitor induces extensive apoptosis in *BRAF*^*V600E*^ CRC cell lines

**Fig. 3:**
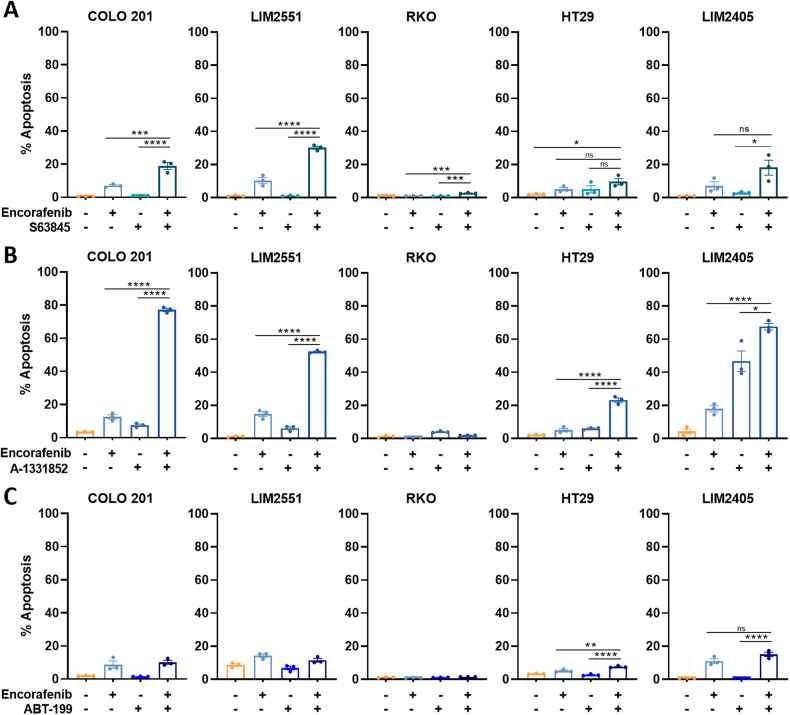
Effect of combining BRAF inhibitors with BH3 mimetics on apoptosis in *BRAF*^*V600E*^ CRC cell lines. *BRAF*^*V600E*^ CRC cell lines were treated with encorafenib (100 nM) alone and in combination with either (**A**) the MCL-1 inhibitor S63845 (1 μM), (**B**) the BCL-X_L_ inhibitor A-1331852 (10 nM) or (**C**) the BCL-2 inhibitor ABT-199 (1 μM) for 72 h and apoptosis induction determined by PI staining and FACS analysis. Values shown are mean ± SEM from a representative experiment performed in technical triplicates. Differences between groups were compared using one-way ANOVA with Tukey’s multiple comparison testing; ns (not significant), *(*p* ≤ 0.05), ***(*p* ≤ 0.001) and ****(*p* ≤ 0.0001).

To determine if the high basal levels of MCL-1 and BCL-X_L_ in CRC cells may restrict their sensitivity to BRAF inhibitor (encorafenib) induced apoptosis, we tested the effect of combining encorafenib with BH3 mimetics targeting either MCL-1 (S63845), BCL-X_L_ (A-1331852), or BCL-2 (ABT-199) on apoptosis in *BRAF*^*V600E*^ CRC cell lines. Combining encorafenib with an MCL-1 inhibitor induced only a modest increase in apoptosis, surpassing 20% in only one (LIM2551) of the five cell lines tested (Fig. 3A). Comparatively, combination treatment of encorafenib with a BCL-X_L_ inhibitor enhanced apoptosis to >50% in 3/5 *BRAF*^*V600E*^ cell lines (Fig. 3B). Finally, consistent with the low levels of BCL-2 expression in *BRAF*^*V600E*^ CRC cells and thus their likely independence on BCL-2 for survival, combination treatment with encorafenib and a BCL-2 inhibitor failed to enhance apoptosis to >25% in any of the cell lines (Fig. 3C).

Notably, HT29 and RKO cells were refractory to all BH3 mimetic + encorafenib combinations. While this may be due to the relatively low level of encorafenib-induced expression of pro-apoptotic proteins (e.g BIM) in these lines (Fig. 1D), examination of basal mRNA expression of pro-survival factors revealed that RKO cells also expressed higher levels of *BCL2* and *BCL2A1* relative to the other cell lines, which may also contribute to its relative drug resistance (Supplementary Fig. 3).

Finally, to confirm these findings in longer term assays, we assessed the efficacy of these drug combinations in clonogenic survival assays in LIM2551 cells over 2 weeks. Consistent with the findings of the apoptosis assays, combining low-dose encorafenib with the BCL-X_L_ inhibitor A-1331852 further reduced colon forming capacity in LIM2551 cells, whereas combining encorafenib with S63845 or ABT-199 had minimal additional effect (Supplementary Fig. S4A, B).

These findings reveal that combining a BRAF inhibitor with a BCL-X_L_ inhibitor is a highly effective means of inducing apoptosis in *BRAF*^*V600E*^ CRC cells.

### BIM is required for BRAF + BCL-X_L_ inhibitor-induced apoptosis

**Fig. 4:**
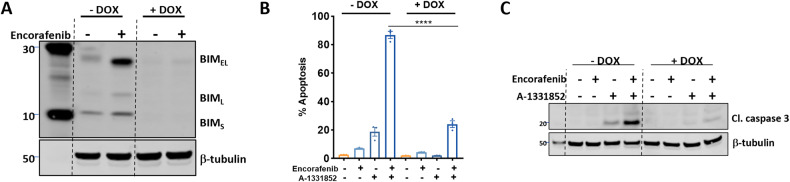
BRAF + BCL-X_L_ inhibitor induced apoptosis requires BIM induction. **A** Validation of CRISPR-mediated deletion of BIM in COLO 201 cells. Cells were maintained in 1 μg/mL of doxycycline (+DOX) to induce BIM deletion and subsequently treated with encorafenib (100 nM) for 6 h to induce BIM expression. BIM expression [extra-long (BIM_EL_), Long (BIM_L_) and short (BIM_S_) forms] was assessed by western blot with β-tubulin used as a loading control. **B** Control and BIM-deleted COLO 201 cells were treated with encorafenib (100 nM) and A-1331852 (10 nM) alone and in combination for 72 h and apoptosis determined by PI staining and FACS analysis. Values shown are mean ± SEM from a representative experiment performed in technical triplicate. Similar results were obtained in a second independent experiment. One-Way ANOVA, with Tukey’s multiple comparison testing; ****(*p*≤0.0001). **C** Western blot analysis of cleaved caspase 3 (Cl. Caspase 3) induction following treatment of control and BIM-deleted COLO 201 cells with encorafenib (100 nM) plus A-1331852 (10 nM) for 12 h. β-tubulin was used as a loading control.

As BRAF inhibitor treatment also significantly enhanced BIM expression in *BRAF*^*V600E*^ CRC cells (Fig. 1, Supplementary Fig. 2), we next assessed whether BIM was required for BRAF + BCL-X_L_ inhibitor-induced apoptosis. To address this, we engineered a doxycycline-inducible CRISPR/Cas9 cell line to delete BIM in *BRAF*^*V600E*^ COLO 201 and LIM2551 cells, and confirmed effective deletion of BIM following doxycycline treatment (Fig. 4A/Supplementary Fig. 5A). Deletion of BIM significantly attenuated BRAF + BCL-X_L_ inhibitor-induced apoptosis in COLO201 cells, as assessed by propidium iodide staining (Fig. 4B) and cleaved caspase 3 levels (Fig. 4C). A rescue of the apoptotic effect was also observed in LIM2551 cells albeit to a lesser extent (Supplementary Fig. 5B). While this finding demonstrates a direct role for BIM induction in BRAF + BCL-X_L_ inhibitor-induced apoptosis, it also suggests that other factors are likely to be involved.

### Combining encorafenib with next generation BCL-X_L_ inhibitors additively enhances apoptosis in *BRAF*^*V600E*^ CRC cells in vitro and suppresses tumour growth in vivo

**Fig. 5:**
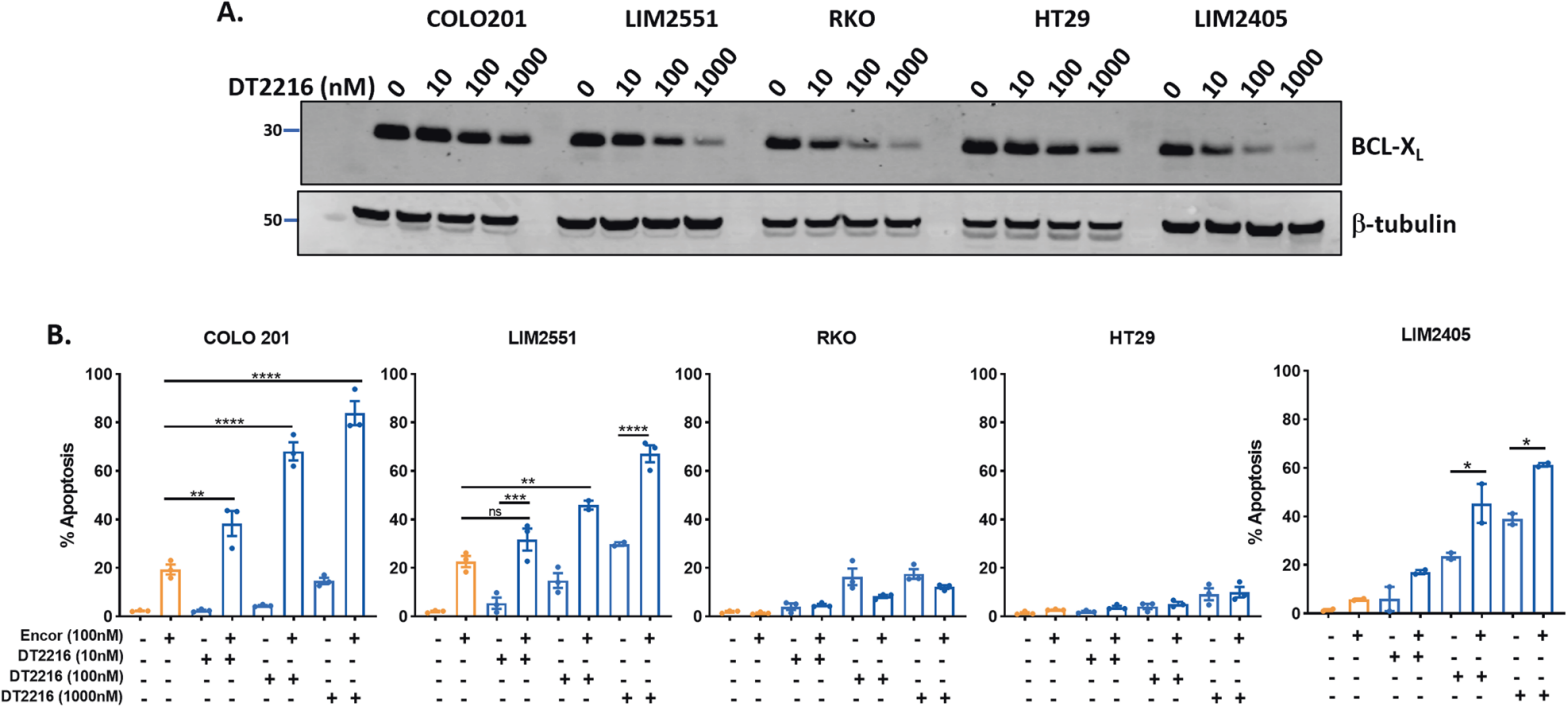
Effect of combining the BCL-X_L_ -targeting PROTAC, DT2216, with encorafenib in *BRAF*^*V600E*^ CRC cells. **A** Western blot analysis of BCL-X_L_ expression following treatment of *BRAF*^*V600E*^ CRC cells with DT2216 (10, 100 and 1000 nM) for 24 h. β-tubulin was used as a loading control. **B** *BRAF*^*V600E*^ CRC cells were treated with encorafenib (100 nM) and DT2216 (10, 100 or 1000 nM) alone and in combination for 72 h and apoptosis determined by PI staining and FACS analysis. Values shown are mean ± SEM from 3 independent experiments, except for LIM2405 (values shown are mean ± SEM from 2 independent experiments). Differences were compared using one-way ANOVA with Tukey’s multiple comparison testing; ns (not significant), *(*p* ≤ 0.05), **(*p* ≤ 0.01), ***(*p* ≤ 0.001), and ****(*p* ≤ 0.0001).

While our findings demonstrate that combining BCL-X_L_ inhibitors with BRAF inhibitors robustly induces apoptosis in *BRAF*^*V600E*^ CRC cells in vitro, the clinical use of BCX-X_L_ inhibitors is currently limited by their induction of thrombocytopenia [30], an on-target toxicity driven by the high dependency of platelets on BCL-X_L_ for their survival [31]. Therefore, we sought to determine the efficacy of combining BRAF inhibitors with two recently emergent strategies for BCL-X_L_ inhibition, designed to circumvent this limitation. First, we utilized DT2216, a proteolysis-targeting chimera (PROTAC) to target BCL-X_L_ for degradation. DT2216 is a conjugate of the BH3 mimetic ABT263 and a ligand for the Von Hippel-Lindau (VHL) E3 ubiquitin ligase, which has minimal expression in platelets [32]. The capacity of DT2216 to target BCL-X_L_ for degradation in a dose-dependent manner was validated in the panel of five *BRAF*^*V600E*^ cell lines (Fig. 5A). Consistent with previous results, combinatorial treatment of DT2216 with encorafenib significantly enhanced apoptosis in a dose-dependent manner compared to either agent alone in COLO201, LIM2551 and LIM2405 cells whilst RKO and HT29 remained refractory to treatment (Fig. 5B).

**Fig. 6:**
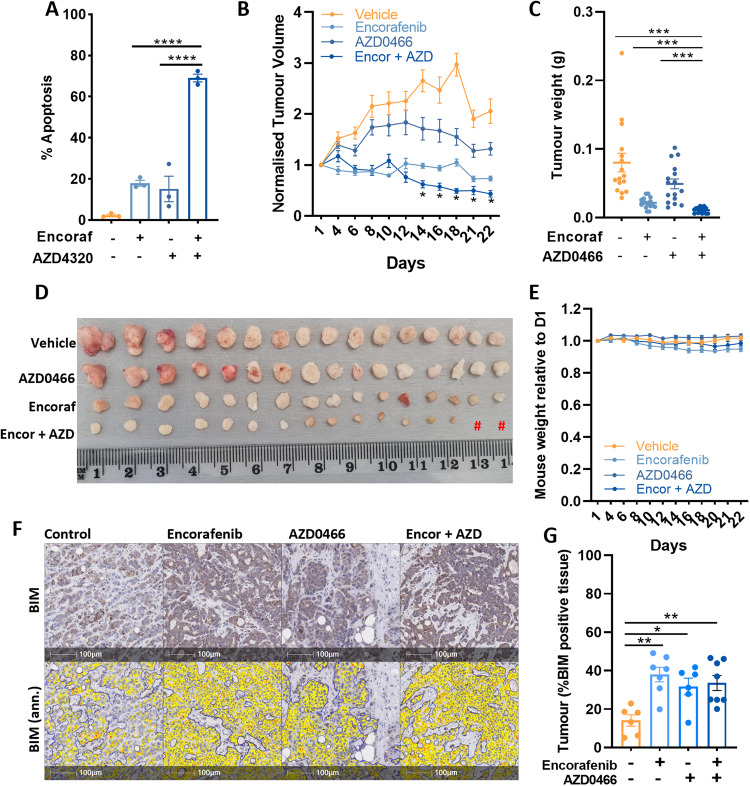
Anti-tumour effects of encorafenib plus AZD4320/AZD0466 on *BRAF*^*V600E*^ CRC cells in vitro and in vivo. **A** COLO 201 cells were treated with encorafenib (100 nM) and AZD4320 (500 nM) alone and in combination for 72 h in vitro and apoptosis determined by PI staining and FACS analysis. Values shown are mean ± SEM from 3 independent experiments. Differences were compared using one-way ANOVA with Tukey’s multiple comparison testing; ****(*p* ≤ 0.0001). **B** Effect of encorafenib and AZD0466 on the growth of COLO 201 xenografts. NOD scid gamma mice (*n* = 8 per group) were subcutaneously injected with 2 × 10^6^ COLO 201 cells into the left and right flanks (*n* = 16 tumours per group except the combination treatment group where *n* = 14). Mice were then randomised to receive either vehicle control, encorafenib (20 mg/kg, b.i.d, og), AZD0466 (103 mg/kg, once weekly, I.V) or the combination, for 22 days. Tumour volume was determined by caliper measurements every second day and normalised to the volume at day 1 of treatment. Values shown are mean ± SEM. (**C**) Weight (g) and (**D**) representative images of excised tumours at experimental endpoint. Hash (#) depicts one less mouse (two less tumours) for the combination treatment group. **E** Relative change in mouse body weight from treatment commencement. Values shown are mean ± SEM. **F** Representative images of immunohistochemical staining of BIM in resected tumours and corresponding annotation performed using HALO software depicting areas of low (yellow), moderate (orange) and strong (red) BIM staining. **G** Quantification of BIM staining (percentage of positively stained tumour cells) in resected tumours. Values shown are mean ± SEM. Differences were compared using one-way ANOVA with Tukey’s multiple comparison testing; *(*p*≤0.05), **(*p*≤0.01), ***(*p*≤0.001) and ****(*p*≤0.0001).

Second, we determined the apoptotic effect of combining encorafenib with the recently developed BCL2/BCLXL inhibitor AZD4320 [33], and its drug dendrimer conjugate AZD0466, designed to minimise toxicity by gradually releasing AZD4320 by hydrolysis thus resulting in lower peak plasma levels, as well as through increased dendrimer retention within the tumour [21]. Combination treatment of COLO 201 cells with encorafenib and AZD4320 in vitro, significantly enhanced apoptosis compared to either agent alone (Fig. 6A). To determine the efficacy of this combination in vivo, COLO 201 cells were grown as xenografts and mice treated with encorafenib (20 mg/kg, bid, oral gavage) and AZD0466 (103 mg/kg, once weekly, tail vein injection) alone or in combination for 22 days. The combination significantly suppressed tumour growth compared to vehicle control or either agent alone and also resulted in tumour regression from treatment commencement (Fig. 6B–D). No significant change in body weight was observed with combination treatment over the duration of the experiment (Fig. 6E), collectively demonstrating that combining a BRAF inhibitor with the BCL-X_L_ inhibitor AZD0466 may be an effective and tolerable treatment for *BRAF*^*V600E*^ CRC. Consistent with our in vitro findings, immunohistochemical staining of resected tumours revealed an increase in BIM expression in mice treated with Encorafenib alone or in combination with AZD0466 (Fig. 6F, G). Somewhat surprisingly, analysis for apoptosis using TUNEL staining failed to reveal a significant difference between treatment groups, which may be due to tumour resection occurring 72-h post the final AZD0466 treatment, a period in which apoptotic cells may have been cleared from the tumour (data not shown). Collectively these findings demonstrate that combining a BRAF inhibitor with the BCL-X_L_ inhibitor AZD0466 may be an effective and tolerable treatment for *BRAF*^*V600E*^ CRC.

## Discussion

Metastatic *BRAF*^*V600E*^ CRC carries an extremely poor prognosis and is in urgent need of better treatments. In this regard, a recent breakthrough was the approval of encorafenib plus cetuximab for treatment of chemorefractory *BRAF*^*V600E*^ metastatic CRC patients [13]. However, objective responses only occur in ~20% of patients and overall survival is only increased by ~4 months, warranting the search for strategies to enhance the efficacy of this treatment regime. Herein, we demonstrate that a limitation of BRAF inhibitors when used either alone or in combination with EGFR inhibitors, is that they fail to effectively induce apoptosis in *BRAF*^*V600E*^ CRC cell lines. Notably, despite their failure to induce apoptosis, we found that BRAF inhibitors induced expression of the pro-apoptotic protein BIM and repressed expression of the pro-survival protein MCL-1, suggesting these agents may prime *BRAF*^*V600E*^ cells to undergo apoptosis.

An important element of this study was the investigation of the mechanism underpinning apoptotic resistance of *BRAF*^*V600E*^ CRC cells to BRAF inhibitors, which was investigated by transcriptomic profiling of basal expression levels of pro-survival genes. This analysis revealed high basal expression of both MCL-1 and BCL-X_L_, and lower expression of BCL-2, BCL-w and BFL1 in all CRCs including the *BRAF*^*V600E*^ subset. Notably, despite the high basal expression of both BCL-X_L_ and MCL-1, a greater enhancement of apoptosis was observed when encorafenib was combined with a BCL-X_L_ inhibitor compared to an MCL-1 inhibitor. While this finding may reflect differences in target inhibition between current BCL-X_L_ and MCL-1 inhibitors, a further explanation may be the capacity of the encorafenib+BCL-X_L_ combination to inhibit both BCL-X_L_ as well as MCL-1, the latter through encorafenib-mediated transcriptional and/or post-translational effects (Fig. 1). Comparatively, when encorafenib is combined with an MCL-1 inhibitor, the apoptotic threshold may remain elevated due to the sustained presence of high BCL-X_L_. In addition, we also demonstrate a key role for BIM induction in encorafenib+BCL-X_L_ inhibitor-induced apoptosis. The encorafenib-induced transcriptional and/or post-translational stabilization of BIM [34–37], and suppression of MCL-1 expression, combined with a BCL-X_L_ inhibitor, may result in sufficient levels of BAX and BAK being released from pro-survival proteins allowing BIM to activate BAX and BAK, and initiate apoptosis [38].

While these findings reveal that combining BRAF and BCL-X_L_ inhibitors represents a promising therapeutic approach for *BRAF*^*V600E*^ CRC, the clinical use of BCL-X_L_ inhibitors is currently limited by their on-target toxicity of thrombocytopenia [30, 31]. We found that combining encorafenib with the BCL-X_L_ degrading PROTAC DT2216 and AZD0466, a novel BCL-2/BCL-X_L_ inhibitor-dendrimer conjugate [21, 39], significantly enhanced apoptosis. Furthermore, combination of encorafenib with AZD0466 significantly reduced tumour growth in vivo and was generally well tolerated in mice. AZD0466 is currently being tested in phase I/II clinical trials for the treatment of haematological malignancies (ClinicalTrials.gov identifier: NCT05205161 and NCT04865419), and our findings suggest that combining encorafenib with AZD0466 may also be a promising approach for treating *BRAF*^*V600E*^ tumours.

The concept of combining MAPK pathway inhibitors with BH3 mimetics has been explored in colorectal cancer and other tumour types. Specifically, a pre-clinical study in *BRAF*^*V600E*^ melanoma demonstrated synergistic induction of apoptosis when BRAF or MEK inhibitors were combined with a MCL-1 inhibitor, which aligned with MCL-1 being the predominant pro-survival protein expressed in melanoma cells [17]. A further study in *KRAS*-mutant NSCLC and other *KRAS*-mutant solid tumour cell lines also reported synergistic cell killing when MAPK pathway inhibitors were combined with BH3 mimetics [40]. Excitingly, these studies have prompted the initiation of a clinical trial of the MEK inhibitor trametinib in combination with the BCL-2/BCL-XL inhibitor navitoclax in patients with advanced or metastatic solid tumours harbouring *KRAS* or *NRAS* mutations (Clinicaltrials.gov identifier: NCT02079740).

The high expression of multiple pro-survival proteins in CRC cells suggests alternative strategies for inducing apoptosis could be through the combined inhibition of BCL-X_L_ and MCL-1. However, pre-clinical studies of these combinations have demonstrated acute liver toxicity, which is consistent with the cooperative role of BCL-X_L_ with MCL-1 in hepatocyte survival [41, 42]. The current approach of using a targeted therapy (BRAF inhibitor) in combination with a BCL-X_L_ inhibitor therefore has the potential to minimize these toxicities, while providing the added benefit of inducing pro-apoptotic proteins such as BIM.

In summary, our findings demonstrate that BRAF inhibitors alone and in combination with the EGFR inhibitor cetuximab fail to induce extensive levels of apoptosis in *BRAF*^*V600E*^ CRC cells. Importantly, we reveal that this can be overcome by combining a BRAF inhibitor with a BCL-X_L_ inhibitor, in both in vitro and in vivo models of *BRAF*^*V600E*^ CRC, suggesting this combination regimen is worthy of clinical validation.

### Reporting summary

Further information on research design is available in the Nature Research Reporting Summary linked to this article.

### Supplementary information

Supplementary Figure Legends

Supplementary Figure 1

Supplementary Figure 2

Supplementary Figure 3

Supplementary Figure 4

Supplementary Figure 5

Original Data

Reporting Summary

## Data Availability

Raw data for this study were generated at the Olivia Newton-John Cancer Research Institute, Melbourne, Australia. The data/datasets generated during and/or analysed during the current study are available from the corresponding author on reasonable request.
