# Supplementary material for: BCL-XL inhibitors enhance the apoptotic efficacy of BRAF inhibitors in BRAFV600E colorectal cancer
Source: Cell Death Dis. 2024 Mar 1;15(3):183. doi: 10.1038/s41419-024-06478-z (PMC10907349; doi:10.1038/s41419-024-06478-z)
Supplement: Supplementary file 1 — Supplementary Figure Legends [file 41419_2024_6478_MOESM1_ESM.docx]

**Supplementary Figure Legends**

**Supplementary Figure 1.** *BRAF^V600E^* CRC cell lines were treated with vemurafenib (Vem, 5 μM) or encorafenib (Enc, 100 nM) for 72 hours and stained with propidium iodide (PI) and analysed by FACS analysis to determine percentage of cells in GO-G1 phase. Values shown are mean ± SEM from a representative experiment performed in technical triplicate. Similar results were obtained in 2 additional independent experiments. Groups were compared using an unpaired Student’s t-test using Welch’s correction; **(*p*≤0.01), ***(*p*≤0.001) and ****(*p*≤0.0001).

**Supplementary Figure 2. Effect of BRAF inhibition on mRNA expression of components of the intrinsic apoptosis pathway.** *BRAF*^V600E^ CRC cell lines (n=11) were treated with vemurafenib (5 μM) for 6-hours and gene expression changes assessed using Affymetrix microarrays. Data shown are mean ± SEM of the relative change in gene expression components of the intrinsic apoptotic pathway. Groups were compared using a two-tailed paired Student’s t-test; *(*p*≤0.05), **(*p*≤0.01), ***(*p*≤0.001) and ****(*p*≤0.0001).

**Supplementary Figure 3. RKO cells contain high basal levels of all pro-survival factors.** Basal mRNA expression of *BCL2L1*, *MCL1*, *BCL2*, *BCL2L2* and *BCL2A1* in 5 *BRAF^V600E^* CRC cell lines as determined by RNA sequencing analysis.

**Supplementary Figure 4. Effect of encorafenib plus BH3-mimetics on clonogenic survival.** (**A/B**) LIM2551 cells were treated with encorafenib (1 nM and 100 nM) and either A-1331852 (10 nM), S63845 (1 μM) or ABT-199 (1 μM) alone and in combination for two weeks, and clonogenic survival assessed by crystal violet staining. Values shown are mean ± SEM from a representative experiment performed in technical triplicate. Similar results were obtained in an additional independent experiments. Groups were compared using One-way ANOVA with Tukey’s multiple comparisons; ns (not significant), *(*p*≤0.05), and ** (*p*≤0.01).

**Supplementary Figure 5. BRAF+BCL-X_L_ inhibitor induced apoptosis requires BIM induction.** (**A**) Validation of CRISPR-mediated deletion of BIM in LIM2551 cells. Cells were maintained in 1 μG/mL of doxycycline (+DOX) to induce BIM deletion and subsequently treated with encorafenib (100 nM) for 6 hours to induce BIM expression. BIM expression [extra-long (BIM_EL_), Long (BIM_L_) and short (BIM_S_) forms] was assessed by western blot with β-tubulin used as a loading control. (**B**) Control and BIM-deleted LIM2551 cells were treated with encorafenib (100 nM) and A-1331852 (10 nM) alone and in combination for 72 hours and apoptosis determined by PI staining and FACS analysis. Values shown are mean ± SEM from a representative experiment performed in technical triplicate. Similar results were obtained in a second independent experiment. One-Way ANOVA, with Tukey’s multiple comparison testing; *(*p*≤0.05).
