# Supplementary figures and images for: BCL-XL inhibitors enhance the apoptotic efficacy of BRAF inhibitors in BRAFV600E colorectal cancer
Source: Cell Death Dis. 2024 Mar 1;15(3):183. doi: 10.1038/s41419-024-06478-z (PMC10907349; doi:10.1038/s41419-024-06478-z)

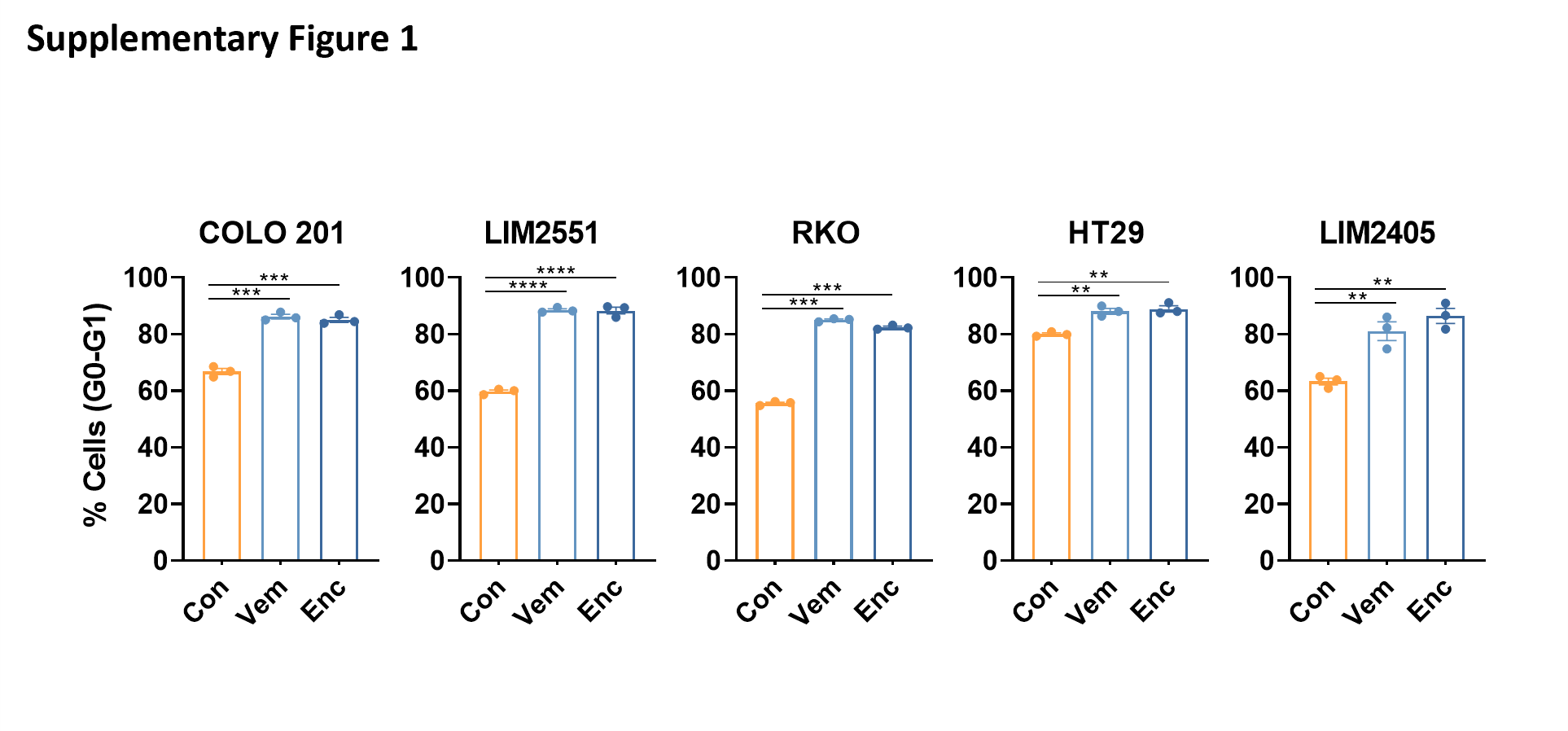

Supplement: Supplementary file 2 — Supplementary Figure 1 [file 41419_2024_6478_MOESM2_ESM.png]

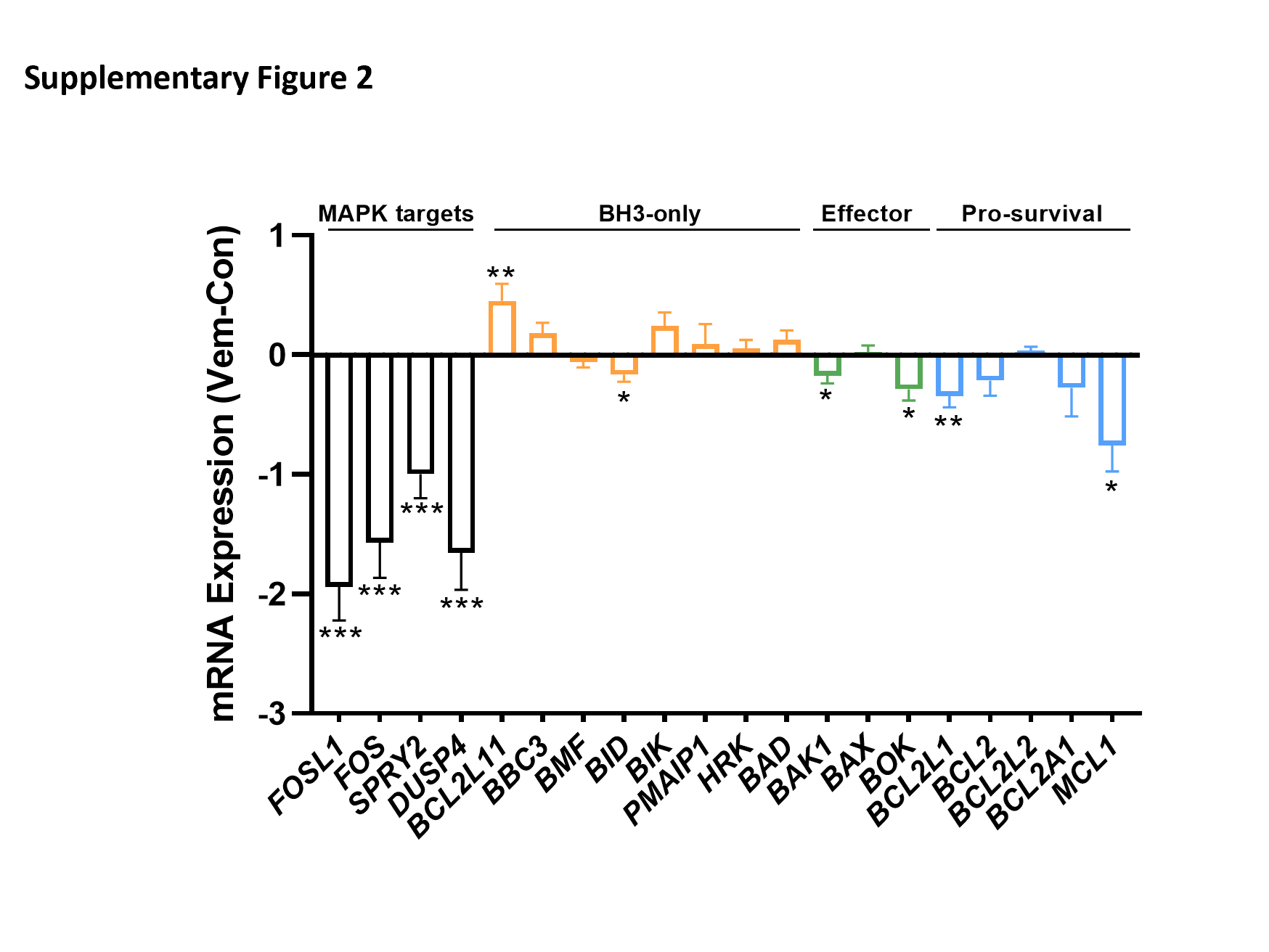

Supplement: Supplementary file 3 — Supplementary Figure 2 [file 41419_2024_6478_MOESM3_ESM.png]

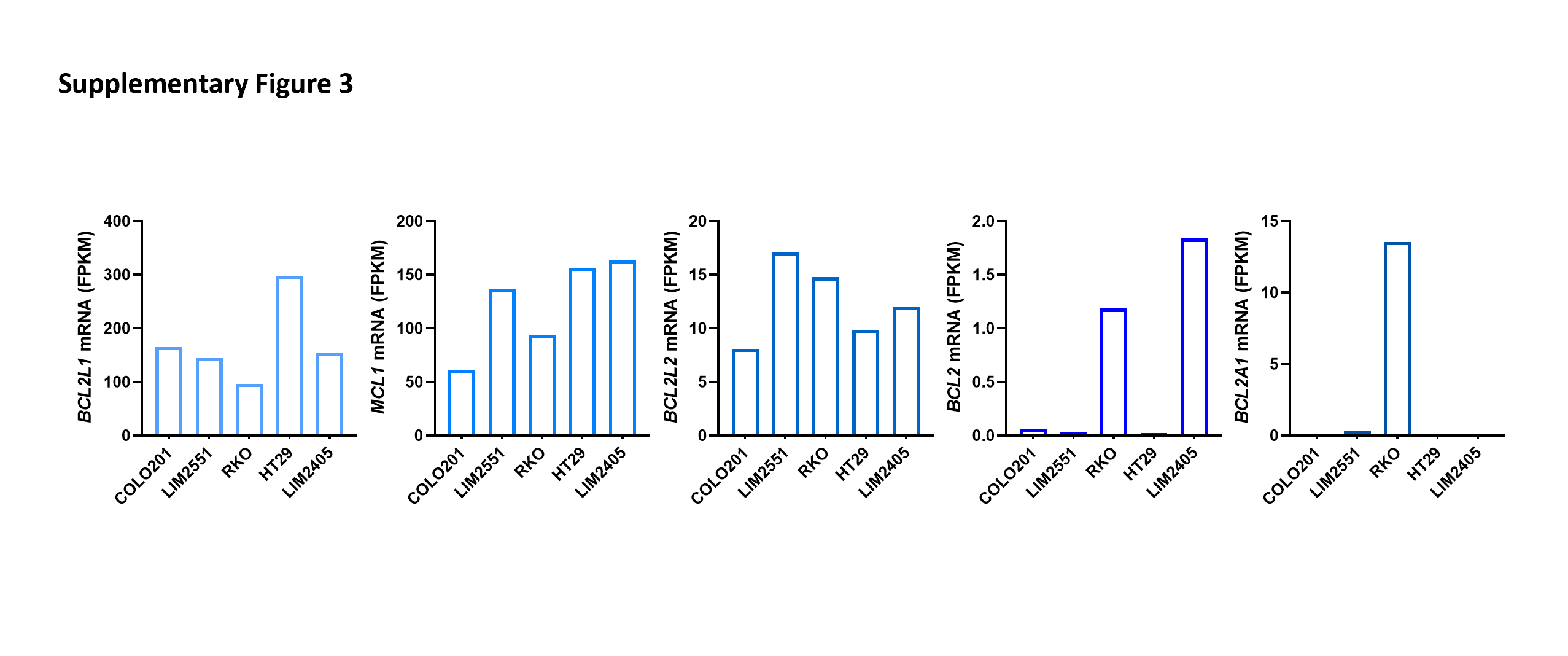

Supplement: Supplementary file 4 — Supplementary Figure 3 [file 41419_2024_6478_MOESM4_ESM.png]

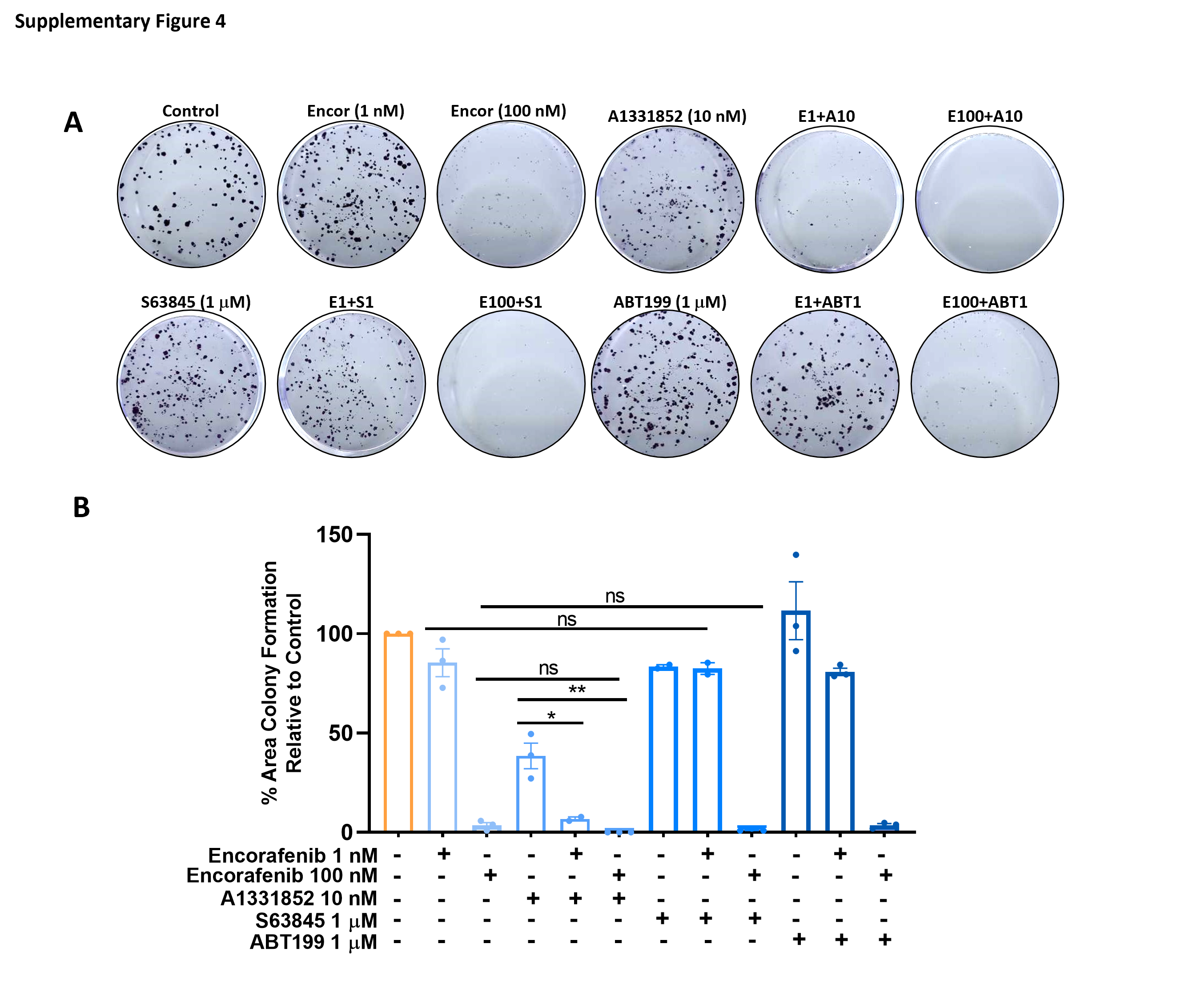

Supplement: Supplementary file 5 — Supplementary Figure 4 [file 41419_2024_6478_MOESM5_ESM.png]

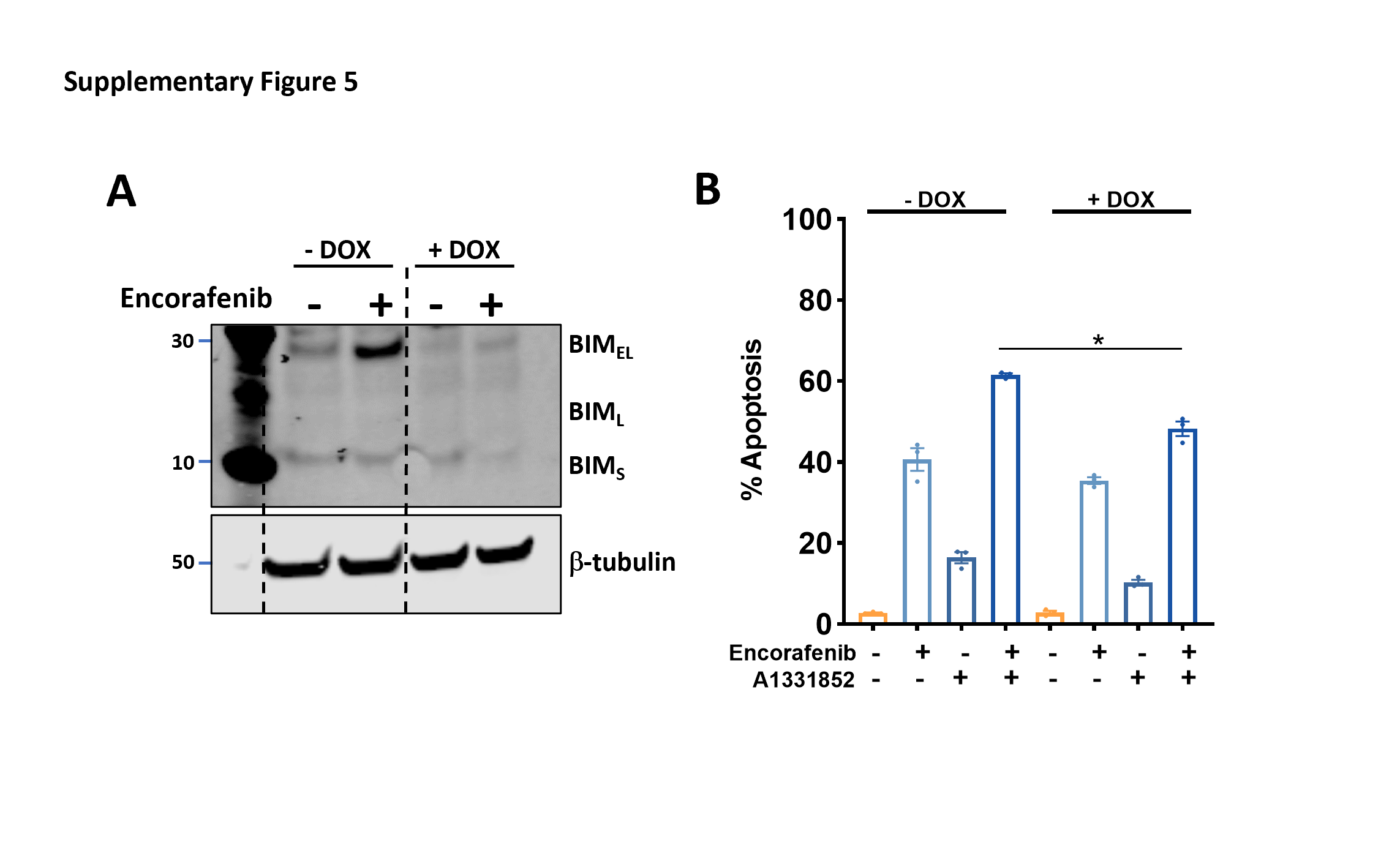

Supplement: Supplementary file 6 — Supplementary Figure 5 [file 41419_2024_6478_MOESM6_ESM.png]
