# Supplementary material for: BCL-XL inhibitors enhance the apoptotic efficacy of BRAF inhibitors in BRAFV600E colorectal cancer
Source: Cell Death Dis. 2024 Mar 1;15(3):183. doi: 10.1038/s41419-024-06478-z (PMC10907349; doi:10.1038/s41419-024-06478-z)

Figure 1D – Full size western blot

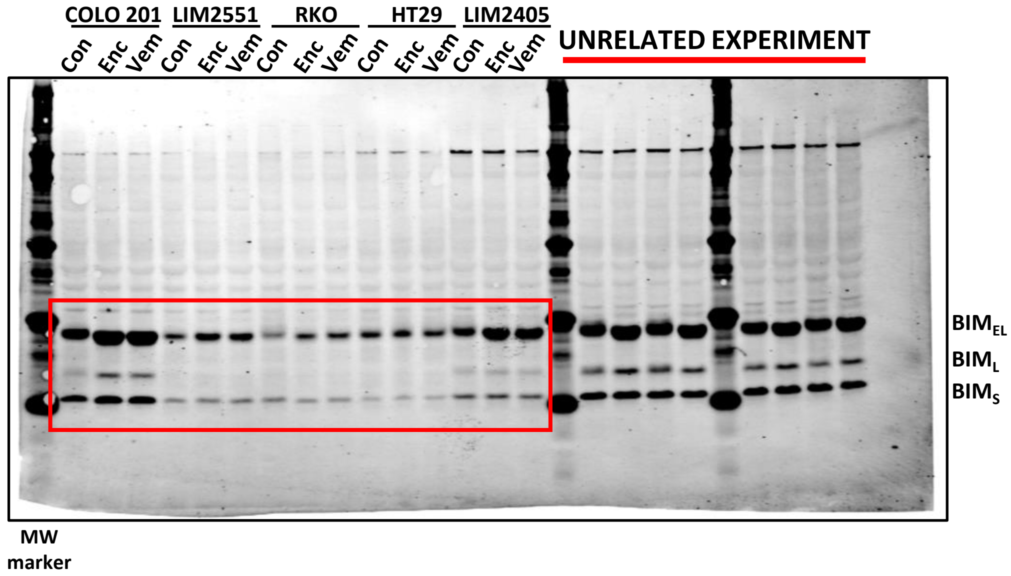

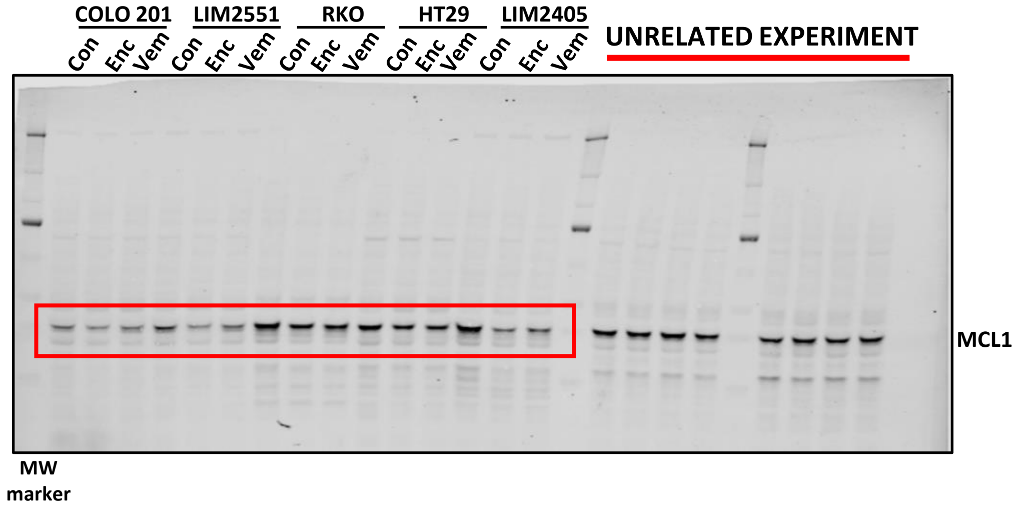

Figure 1D – Full size western blot

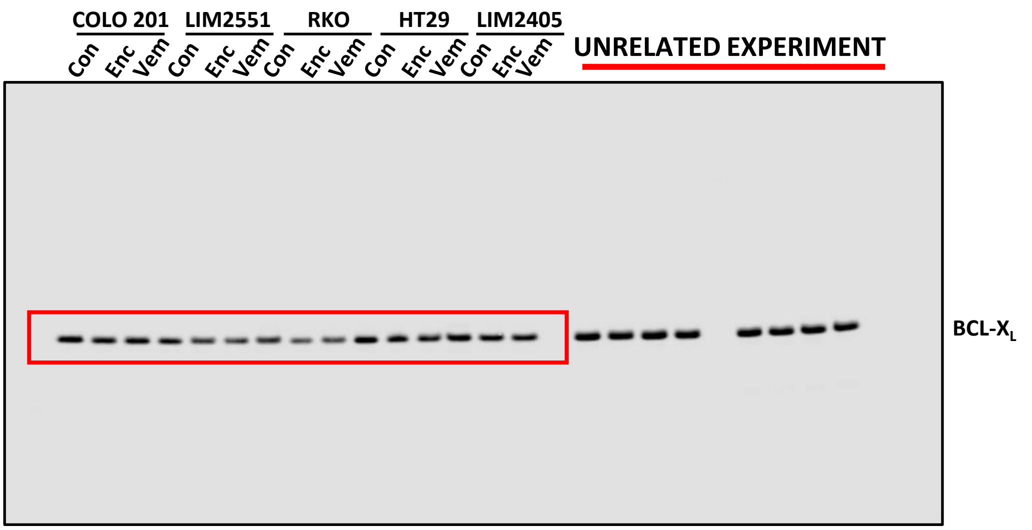

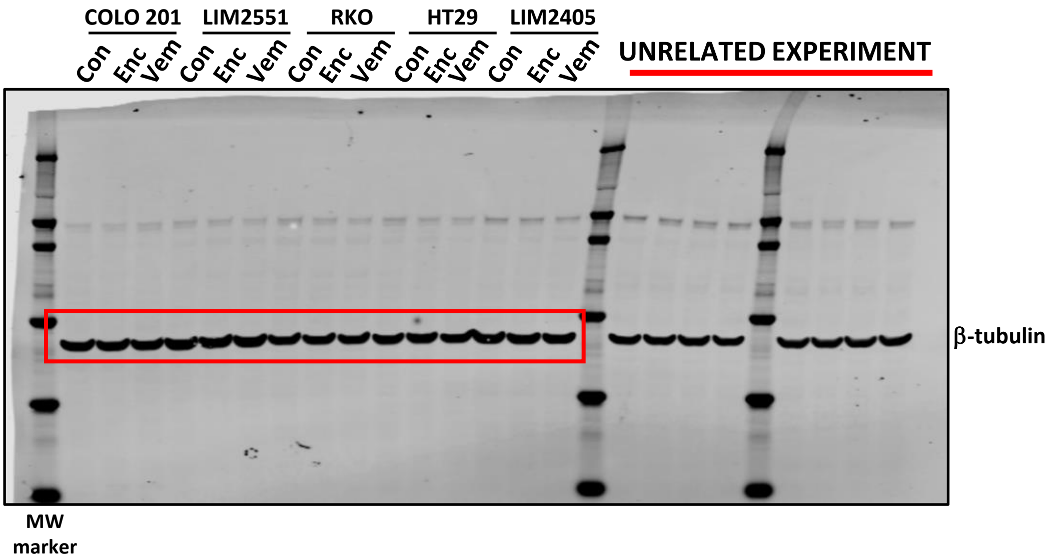

Figure 1E – Full size western blot

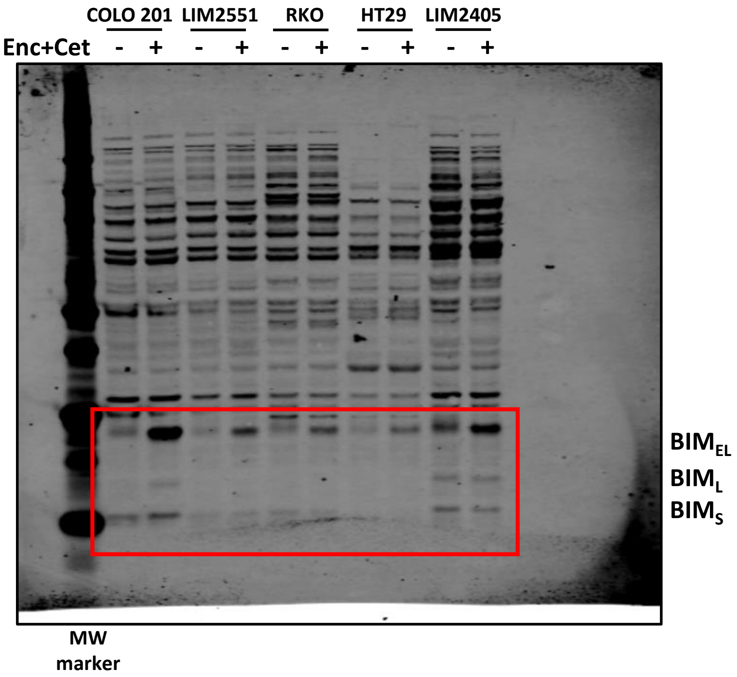

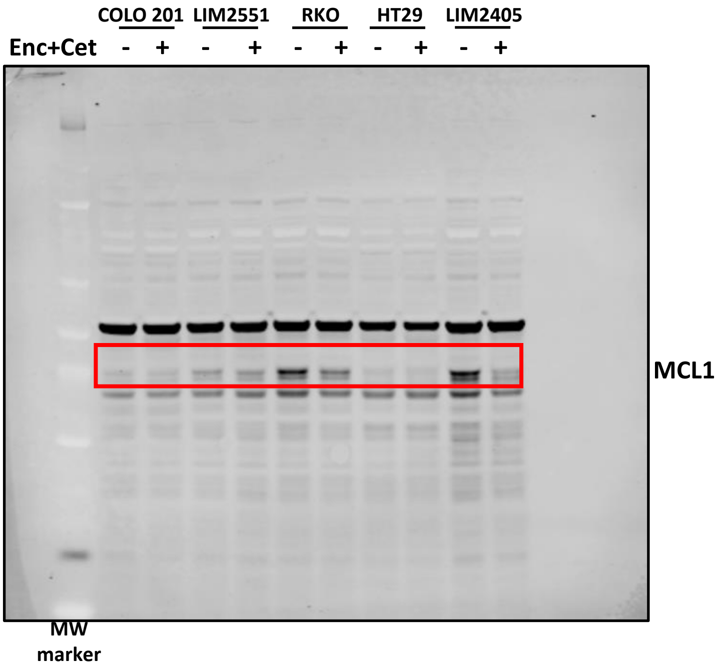

Figure 1E – Full size western blot

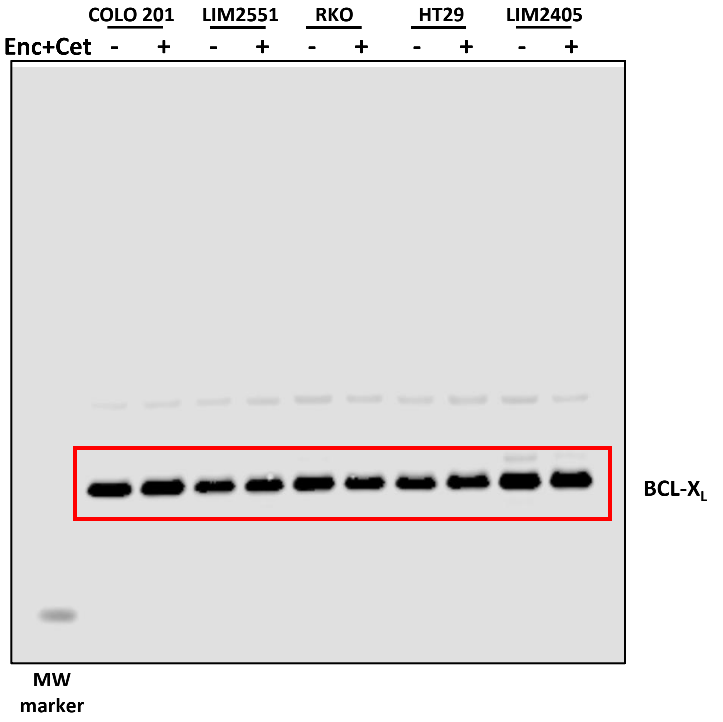

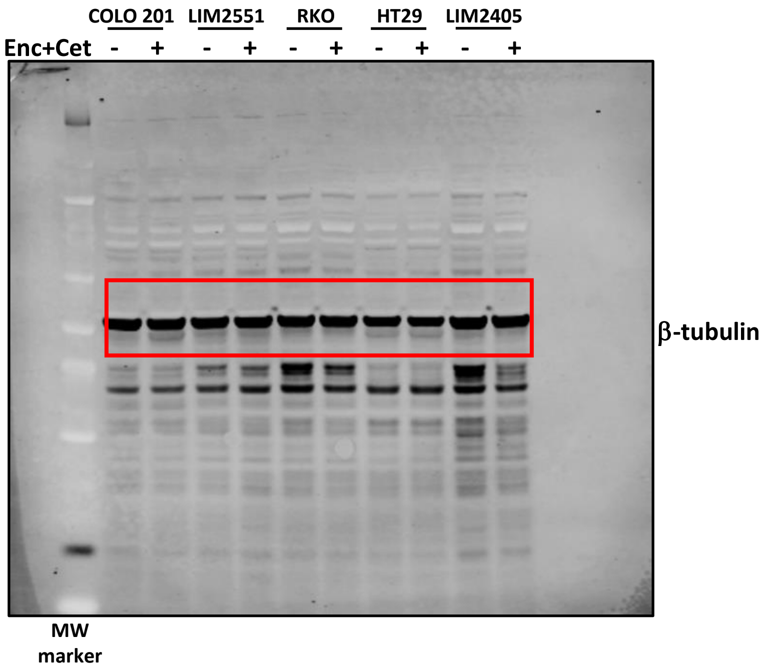

Figure 4A – Full size western blot

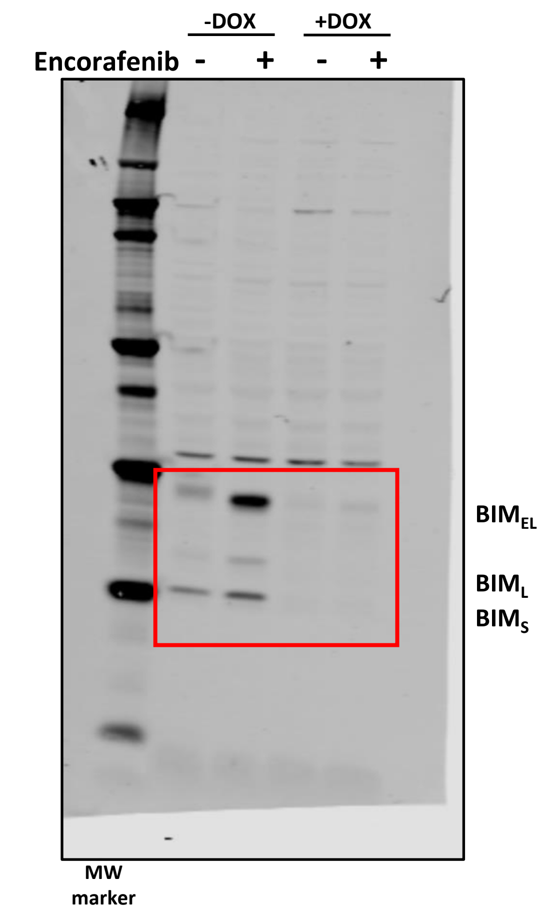

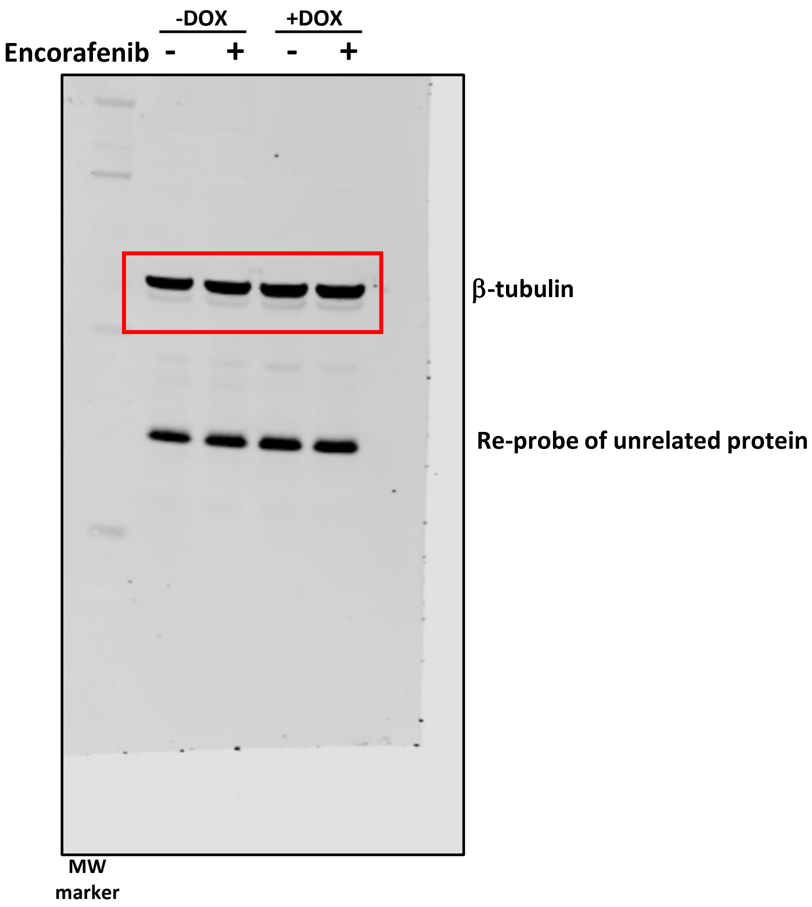

Figure 4C – Full size western blot

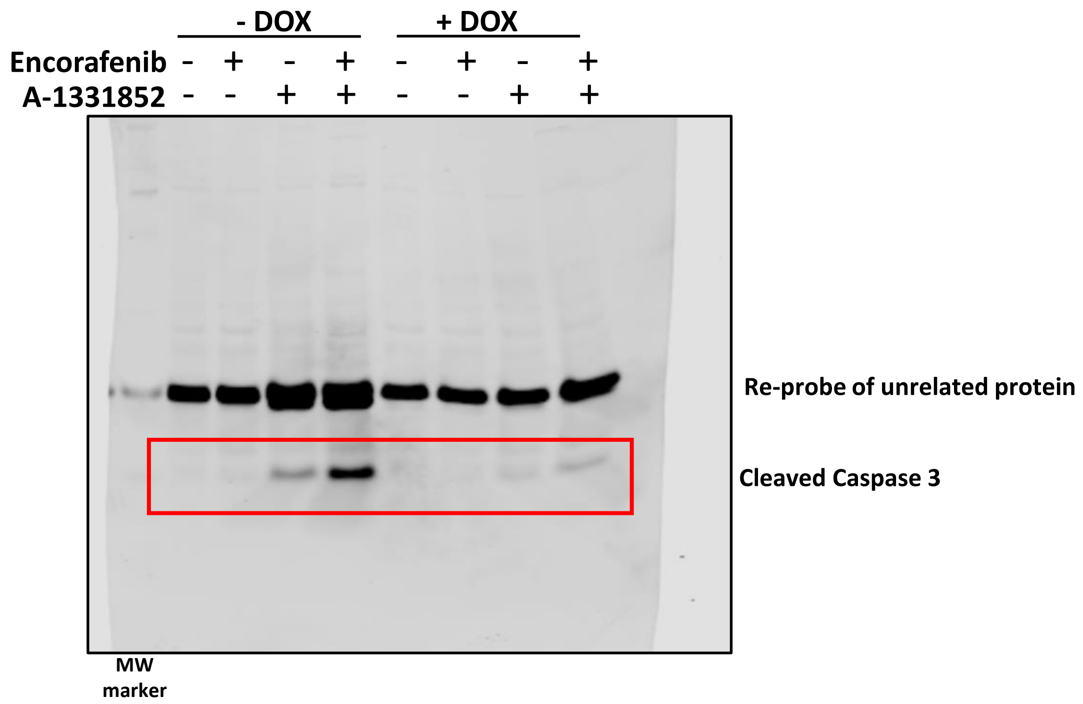

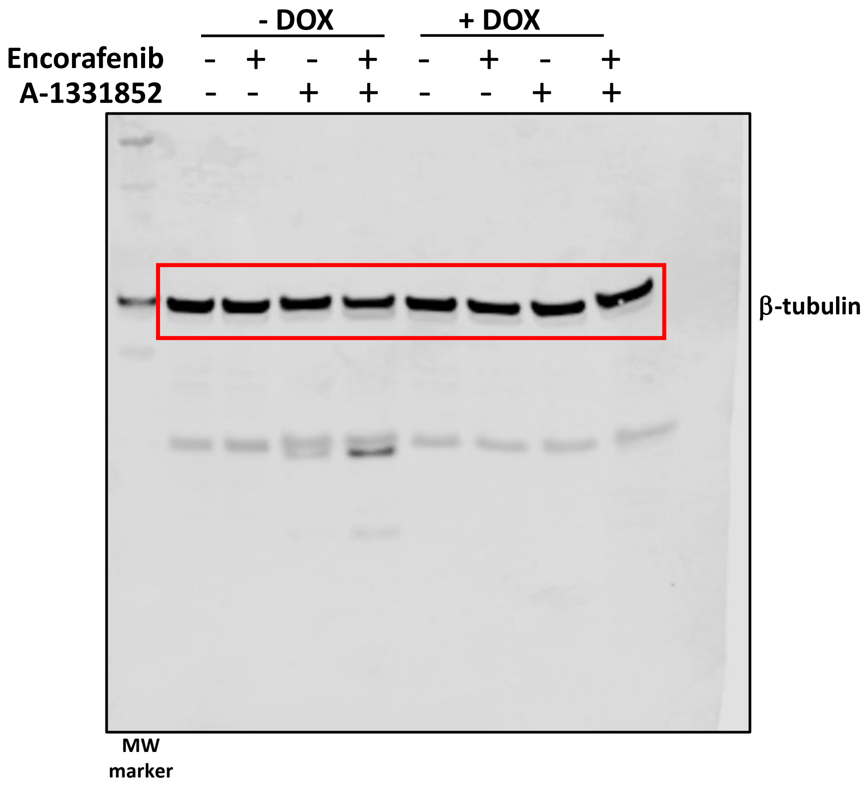

Figure 5A – Full size western blot

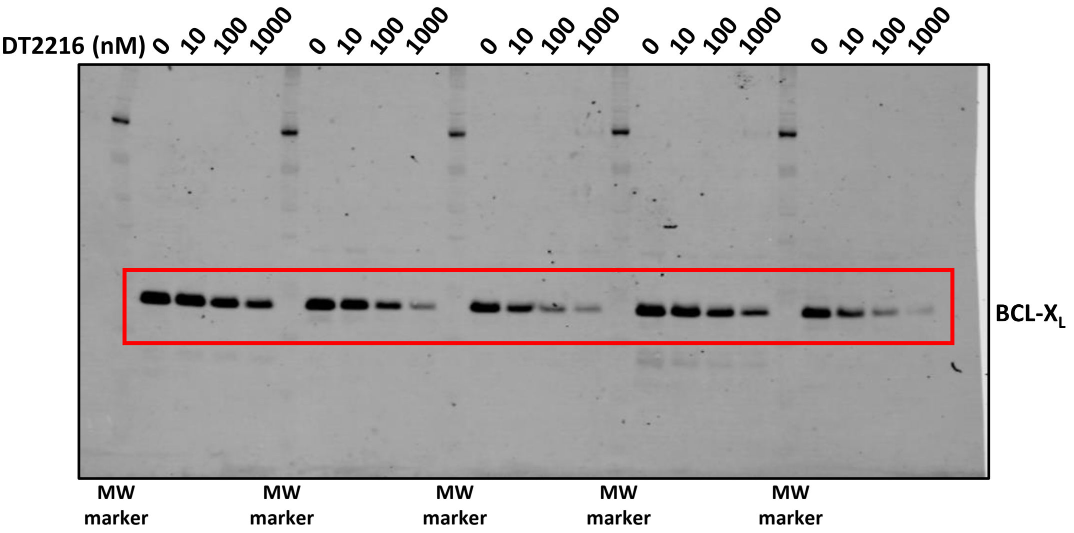

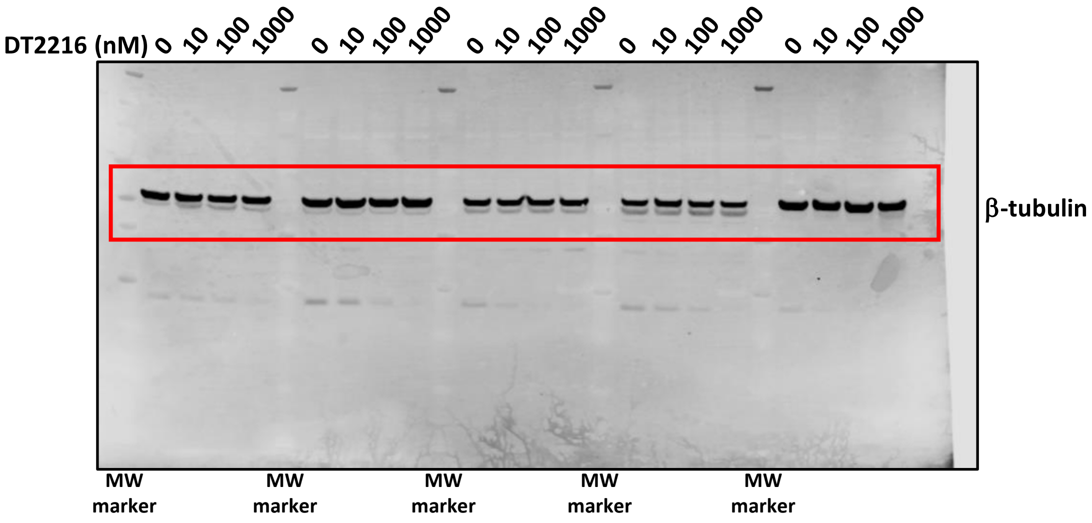

Supplementary Figure 5A – Full size western blot

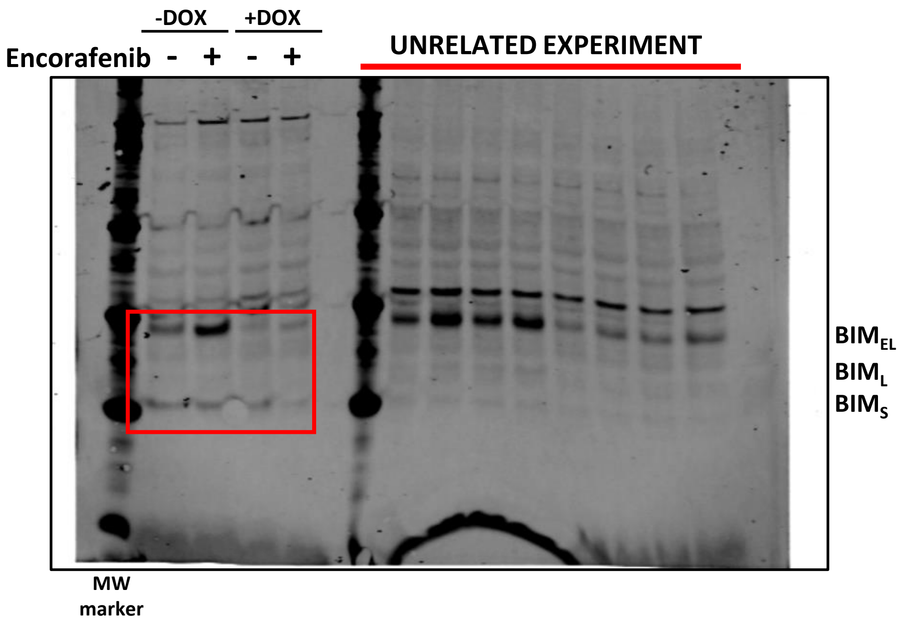

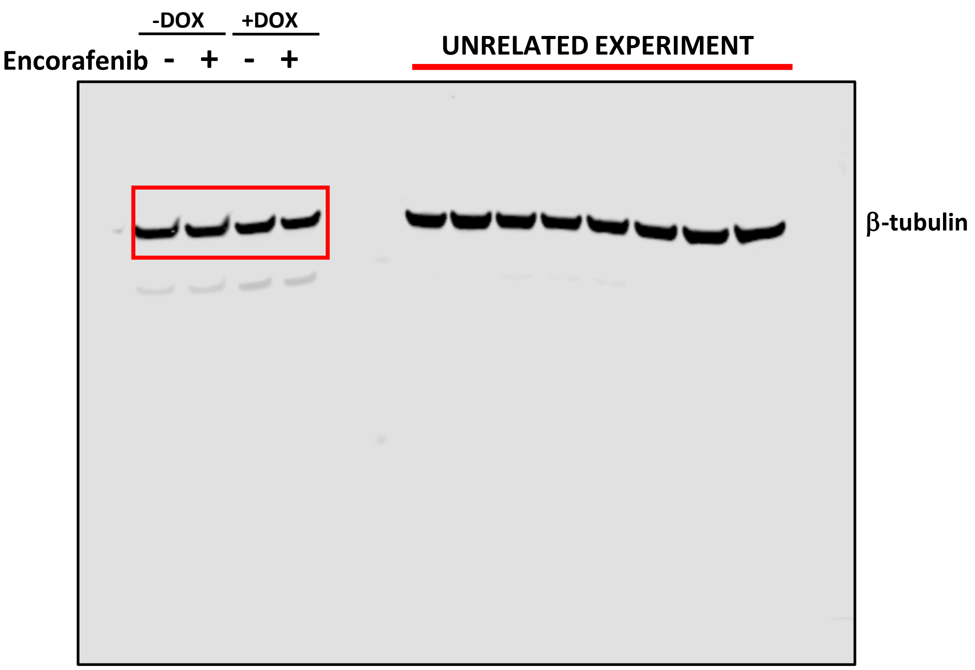

Supplement: Supplementary file 7 — Original Data [file 41419_2024_6478_MOESM7_ESM.pdf]
